# The effect of EFL learners’ identity on English speaking proficiency and autonomous learning skills among university students: the mediating role of speaking anxiety

**DOI:** 10.3389/fpsyg.2026.1773756

**Published:** 2026-04-10

**Authors:** Yeqing Tao, Mengyun Xiao, Samah Ali Mohsen Mofreh, Sultan Salem

**Affiliations:** 1 School of Foreign Languages, Shaoguan University, Shaoguan, China; 2 School of Educational Studies, Universiti Sains Malaysia (USM), Penang, Malaysia; 3 Department of Economics, Birmingham Business School, College of Social Sciences, University of Birmingham, Birmingham, United Kingdom

**Keywords:** autonomous learning skills, EFL learner identity, speaking anxiety, speaking proficiency, university students

## Abstract

**Introduction:**

As a Foreign Language (EFL) learner identity constitutes a pivotal sociopsychological factor in higher education; however, empirical research on how learner identity relates to speaking proficiency (SP) and autonomous learning skills (ALS) remains limited, particularly regarding the affective mechanism through speaking anxiety (SA).

**Methods:**

Drawing on Identity Theory and the Affective Filter Hypothesis, the present study examined the effect of EFL learner identity, modeled as a second order latent construct indicated by identity belongingness (IDB) and identity expectations (IDE), on SP and ALS among 392 Chinese non-English major sophomores, and tested speaking anxiety (SA) as a mediator using structural equation modeling (SEM).

**Results:**

The results showed that overall learner identity significantly predicted both SP and ALS, and that SA served as a meaningful affective pathway, accounting for approximately 36% of the total effects on learning outcomes.

**Discussion:**

These findings highlight the importance of fostering an identity-supportive learning environment that reduces the “affective filter” and strengthens university students’ oral communication and learner autonomy in EFL contexts.

## 1 Introduction

As globalization and digital transformation continue, improving English-speaking skills has become a main goal in university-level English education (Khoso et al., 2025). For Chinese university students learning English as a foreign language, speaking well is important not just for class participation and academic work, but also for internships, job opportunities, and communicating across cultures. Even after years of education reform, many students especially those who are not English majors struggle to speak English. This is often called “deaf and dumb English” (Shi et al., 2024; Xu et al., 2022). The focus on exams in teaching also leads to more students experiencing speaking anxiety, which can hurt their fluency, accuracy, complexity, and willingness to speak. Finding ways to help students who can learn but are still hesitant or afraid to speak remains a major challenge for English education in China.

Recently, the idea of learner identity has offered a new way to understand why students differ in motivation, feelings, and behavior during language learning (Khoso et al., 2025). Learner identity is not fixed. Instead, it is an ongoing process in which people shape and rethink their sense of “who I am” and “who I can become” across different social and cultural settings (Gu, 2010). Factors such as school systems, interactions with classmates, relationships with teachers, and broader social views all shape identity. These factors affect students’ engagement, the strategies they use, and their persistence. When students see themselves as real English users, they usually feel more confident and are more willing to speak up (Khoso et al., 2025; Taleb Falah and Mohammed, 2025). This sense of ownership often leads to better speaking skills in real-life situations.

Speaking Anxiety (SA) is widely seen as an important emotional factor in learning a foreign language. It often involves fear of being judged and a sense of uncertainty. While earlier studies have examined what causes SA, such as teaching methods or language skills (Teimouri et al., 2019), we know less about how SA affects the relationship between a learner’s identity and learning outcomes. This is important because language learning is closely connected to how people see themselves and feel they belong.

There is still much to learn about how identity connects to specific skills. Such as Speaking Proficiency (SP) is usually measured as a single ability, but its parts accuracy, fluency, and flexibility may each be affected differently by identity factors (Wang et al., 2024). In the same way, Autonomous Learning Skills (ALS) are important for long-term success, but their relationship to learner identity remains poorly understood, especially among Chinese students who are not English majors (Chen, 2022).

This study draws on Identity Theory (Stryker and Burke, 2000), which conceptualizes self-concept as shaped by social roles and group membership. In line with this perspective, EFL learner identity is operationalized through two dimensions: Identity Belongingness (IDB), referring to learners’ perceived connection and affiliation with the learning community, and Identity Expectations (IDE), referring to learners’ future oriented goals and expectations associated with the learner role. A more secure and positive learner identity is expected to be associated with lower SA and, in turn, with better speaking proficiency and stronger self-regulation.

In addition, Krashen’s (1982) Affective Filter Hypothesis is used to explain the mediating role of SA. The hypothesis posits that negative affect, including anxiety, can raise the affective filter and hinder language input processing and oral performance. Accordingly, a strong learner identity may reduce the affective filter by alleviating SA, thereby facilitating speaking performance and supporting the confidence and persistence required for autonomous learning.

To fill these research gaps, this study uses Structural Equation Modeling (SEM) to examine how overall EFL learner identity (specified as a second-order construct reflected by IDB and IDE relates to SP (accuracy, fluency, flexibility) and ALS (goal setting, learning strategies, self-management). It also tests whether SA mediates these relationships among Chinese undergraduates who are not English majors. The goal is to provide a clearer, theory-based account of how identity and affect jointly shape learning outcomes and to offer practical implications for creating more supportive EFL learning environments.

## 2 Literature review

### 2.1 Theoretical foundations

This study integrates sociopsychological identity processes with affective and self-regulatory mechanisms to explain EFL learners’ SP and ALS. Identity Theory (Stryker and Burke, 2000) conceptualizes identity as role based self-meaning shaped through social interaction and group membership. In tertiary EFL settings, learner identity can be reflected in both a sense of belongingness to the learner community and future oriented role expectations. Following Tajeddin et al. (2023), the present study models EFL learner identity as a higher order construct indicated by IDB and IDE, allowing identity to be examined as an integrated sociopsychological resource while retaining its two measurement dimensions.

Krashen’s (1982) Affective Filter Hypothesis provides the affective mechanism in the proposed model. It argues that negative affect, particularly anxiety, can constrain language intake, processing, and willingness to communicate. Accordingly, a stronger overall learner identity is expected to reduce speaking anxiety (SA) by strengthening perceived legitimacy and security in the learner role, thereby lowering the affective filter during oral communication.

**FIGURE 1:**
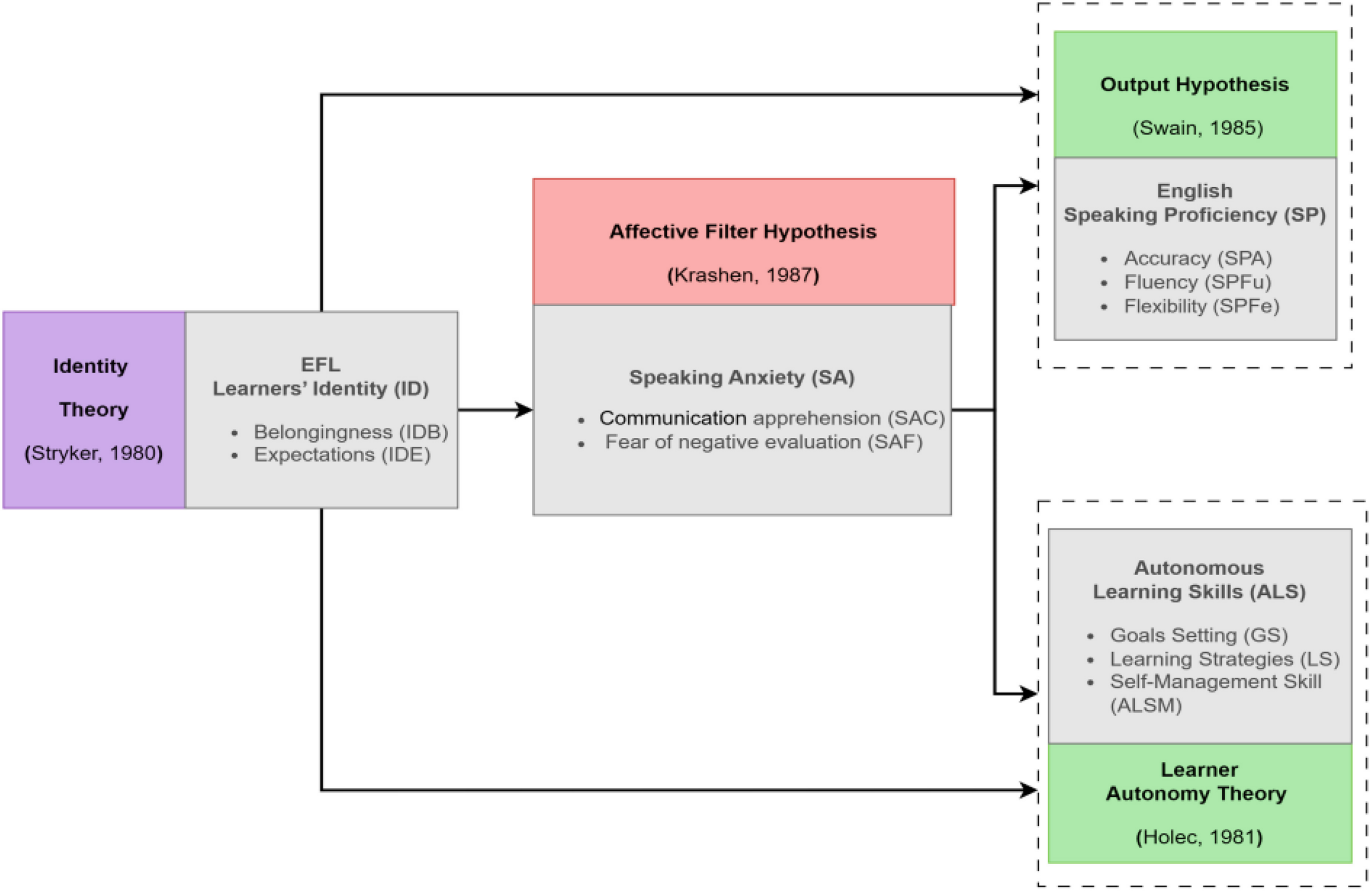
Theoretical framework.

To connect identity to the focal outcomes, Swain’s Output Hypothesis (Swain, 1985, 2005) emphasizes that producing language promotes noticing, hypothesis testing, and self-monitoring. This supports conceptualizing SP as a multidimensional construct encompassing accuracy (SPA), fluency (SPFu), and flexibility (SPFe). The emphasis on noticing and self-monitoring during oral production is also conceptually aligned with the self-regulatory processes underlying ALS. In parallel, Learner Autonomy Theory (Holec, 1981) frames ALS as learners’ capacity to set goals, employ strategies, and manage learning processes. Together, these perspectives justify a model in which overall learner identity predicts SP and ALS both directly and indirectly through SA. Figure 1 summarizes the proposed theoretical framework.

### 2.2 EFL learners’ identity and English-speaking proficiency

Research shows that EFL learners’ identity is important for how they engage with English. Xiao and Zhao (2022) found that Chinese learners’ self-concept and commitment to learning are closely linked to their identity, which varies across different social settings. Peng (2023) also described learner identity as a dynamic construct shaped by cognitive factors, opportunities, and personal choices. Together, these studies show that a learner’s sense of self is shaped by social interactions and directly affects their motivation and language-learning strategies.

Although many studies have looked at the link between learner identity and language learning, there is still little research on how learner identity affects the development of English SP. Some studies, such as Shen (2022), have examined psychological factors, such as mindfulness and resilience, in addressing language learning anxiety, but few have focused on how identity influences oral performance. Most research instead looks at broader topics like self-perception, language learning strategies, or learner investment, often among English majors or high school students (Dewaele and Ip, 2013).

There is a clear need to examine how the IDB and IDE components of EFL learners’ identity affect speaking performance among non-English-major university students in China. These students often deal with large classes and limited oral practice, which may make psychological factors, such as identity, even more important (Gao et al., 2025; Taleb Falah and Mohammed, 2025). Studying this connection can help fill a gap in current research and offer better understanding of the psychological factors behind oral skill development.

This study aims to fill this gap by examining how the two parts of EFL learners’ identity belongingness and expectations affect speaking performance among non-English-major university students in China. These students often have large classes and few opportunities to practice speaking, which may make identity even more important in their language use. Learning about this link can give a fuller view of the emotional and motivational factors that shape speaking development.

Based on this, the following hypothesis was developed:

*H1*: EFL Learner Identity, modeled as a second-order latent construct, significantly influences English Speaking Proficiency (accuracy, fluency, and flexibility) among non-English-major university students in China.

### 2.3 EFL learners’ identity and autonomous learning skills

Research shows there is a strong connection between learner identity and the development of ALSs. Kashefian-Naeeini et al. (2024) found in a mixed-methods study that a strong learner identity can improve self-directed learning. But external pressures, like exams, can lower motivation and autonomy, suggesting that identity’s role in learning can change. Xuan Mai et al. (2024) examined what non-English-major students think prevents them from participating in English-speaking classes and found that both cognitive and emotional factors affect engagement. Little and Erickson (2015) also noted that identity and autonomy are key components of the European Language Portfolio and are important for learners’ self-assessment and reflection.

Although there have been some insights, researchers have not focused much on how identity affects the different parts of ALS. For example, Lockwood (2016) examined the emotional, spatial, and political dimensions of learner autonomy, demonstrating that autonomy is shaped by both the individual and the wider context. Little (2022) described learner autonomy as a dynamic process in teaching and learning, in which learners plan, carry out, check, and assess their own learning. Still, how learner identity supports engagement and improves specific ALS areas, such as goal setting, strategy use, and self-management, has not been studied much. Chong and Reinders (2025) reviewed 61 studies on learner autonomy and found that researchers define and measure autonomy in various ways. They point out that clearer theories are needed, especially to understand how autonomy and learner identity connect in EFL settings. More research is needed to examine how aspects of learner identity affect autonomous learning skills among non-English-major university students in China.

Based on this, the following is proposed:

*H2:* EFL Learner Identity, modeled as a second-order latent construct, significantly influences autonomous learning skills (goal setting, learning strategies, and self-management skills) among non-English major university students in China.

### 2.4 Speaking anxiety for English learners

SA has long been recognized as an important emotional factor that can strongly influence language learners’ SP and achievement in ALS. Many studies show that anxiety can make it harder for learners to communicate in a foreign language, often reducing their self-confidence, participation, and overall performance.

Anxiety is often considered a major obstacle to learning a new language, especially in speaking. MacIntyre and Gardner (1991) found that language anxiety, particularly during speaking, is linked to lower language performance. Their research shows that anxiety makes it more difficult for learners to speak accurately and fluently, which slows the development of speaking skills. Yildiz (2021) identified five factors that contribute to speaking anxiety among students who are not English majors: English proficiency, self-assessment, learner behaviors, deficiencies, and cultural differences.

Horwitz (2001) also found that speaking anxiety directly affects learners’ confidence and willingness to participate in speaking activities, both of which are important for language improvement. Recent research suggests that anxiety can lower motivation and affect metacognitive strategies, which may lead to a decline in English academic writing skills. Anxiety can cause poor language learning in some people, and there is a consistent moderate negative correlation between anxiety and performance (Horwitz, 2001; Xu and Xie, 2024).

SA has been shown to act as a link between psychological factors, such as learner identity, and language learning outcomes (Demir and Kan, 2025). Research indicates that EFL learners’ sense of identity affects their emotions, such as anxiety, which, in turn, influences their language performance and their participation in independent learning (Jia et al., 2026). For example, learners who see themselves as outsiders to the target-language community often feel more anxious, which makes them less likely to engage in speaking practice (Bashori et al., 2022). This can slow the development of both SP and ALS.

Learner identity has been widely studied for its impact on speaking anxiety. Both identity belonging and identity expectations are important in shaping how learners see themselves within the language community, which in turn affects their anxiety levels (Hashemi, 2011). Learners who feel they belong to the English-speaking community tend to be more confident and less anxious when speaking. In contrast, those with weaker feelings of belonging or unrealistic expectations often feel more anxious, which can lower their participation and performance (Wang et al., 2022). This shows that identity influences not only learners’ emotions but also their speaking anxiety, which affects both speaking performance and autonomous learning strategies. Addressing speaking anxiety in language learning is important, especially to strengthen learners’ identity and encourage their participation in speaking tasks.

When educators understand how identity shapes anxiety, they can create better interventions to help learners feel they belong, reduce anxiety, and improve language performance and independent learning. Based on these findings, the following hypothesis is proposed:

*H3*: Speaking anxiety significantly mediates the relationship between the second-order EFL Learner Identity construct and both SP and ALS.

## 3 Materials and methods

### 3.1 Research design

This study used a quantitative, non-experimental correlational design with a cross-sectional approach to explore how EFL learners’ identity affects English SP and ALSs among university students in China who are not English majors. SA was studied as a mediating variable. This design was chosen because it allows researchers to examine predictive and mediating relationships among psychological factors without altering any variables (Cohen et al., 2018; Creswell, 2014).

### 3.2 Sampling

*A priori* power analysis conducted with G*Power determined a target sample size of 400 college students. A stratified sampling method was used to recruit sophomore non-English majors from public universities in Guangdong Province, China. Stratification was performed by major type, institution, and residential area, and proportional samples were drawn from each stratum to improve representativeness across disciplines and regions. Ultimately, 392 valid questionnaires were collected, closely aligning with the sample size recommended by the power analysis.

The demographic composition included 180 male participants (46.0%) and 212 female participants (54.0%). Of the total, 216 students were from rural areas (55.1%), and 176 were from towns (44.9%). Participants represented diverse majors: Humanities and Social Sciences (142, 36.2%), Engineering (73, 18.6%), Science (18, 4.6%), Medicine (5, 1.3%), Agronomy (3, 0.8%), and Others (151, 38.5%). The sample distribution was sufficiently representative of gender, residential background, and major type. Detailed demographic information is provided in Table 1.

**TABLE 1:** Demographics of participants.

Items	Type	Frequency	Percentage (%)
Gender	Male	180	46.0
	Female	212	54.0
Area	Rural area	216	55.1
	Town	176	44.9
Majors	Humanities and social science	142	36.2
	Engineering	73	18.6
	Science	18	4.6
	Medicine	5	1.3
	Agronomy	3	0.8
	Others	151	38.5

### 3.3 Instruments

#### 3.3.1 Questionnaire on EFL learners’ identity, speaking anxiety, and autonomous learning skills

A questionnaire consisting of three established scales was used to assess the key constructs: EFL learners’ identity, SA, and ALS. To ensure both linguistic and conceptual equivalence in the Chinese context, a rigorous translation and adaptation process was implemented (Brislin, 1986). The original English scales were independently translated into Chinese by two bilingual experts in applied linguistics. Any discrepancies were resolved through discussion, resulting in a reconciled Chinese version. This version was subsequently back-translated into English by a third bilingual expert who was not involved in the initial translation. The back-translated version was compared with the original to confirm semantic consistency, and minor adjustments were made to the Chinese version as needed. The questionnaire was piloted with a sample of 45 non-English majors who were not included in the main study to evaluate clarity, comprehensibility, and time requirements. Based on pilot feedback, minor wording adjustments were made to improve item clarity. The final questionnaire required approximately 10–12 min to complete and utilized a five-point Likert scale ranging from 1 (Strongly Disagree) to 5 (Strongly Agree).

The “EFL Learners’ Identity” scale, adapted from Tajeddin et al. (2023), was designed as a second-order latent construct with two related first-order dimensions: Identity Belongingness (IDB, 6 items) and Identity Expectations (IDE, 5 items). Based on Identity Theory (Stryker and Burke, 2000), this approach reflects both how individuals feel connected to the EFL learner community (belongingness) and their future goals in that role (expectations). Specifying identity as a second order construct allows the SEM to represent identity as an integrated latent variable while preserving its multidimensional measurement structure.

The SA scale, which has 10 items, was adapted from Horwitz et al. (1986) and Aydin et al. (2020). It measures two areas: Communication Apprehension (4 items) and Fear of Negative Evaluation (6 items). The Autonomous Learning Skills (ALSs) scale, also with 10 items, was adapted from Phuong and Tham (2022). It includes three subscales: Goal Setting (3 items), Learning Strategies (4 items), and Self-Management Skills (3 items). For both scales, the items were adjusted to fit the Chinese university context while keeping the original theoretical meaning. Table 2 presents the questionnaire structure (see Supplementary material).

**TABLE 2:** The components of the questionnaire.

Content (dimension)	Subscale	No. of items	Source
EFL learners’ identity	Belongingness 6	11	(Tajeddin et al., 2023)
	Expectations 5		
Speaking anxiety	Communication apprehension 4	10	(Horwitz et al., 1986; Aydin et al., 2020)
	Fear of negative evaluation 6		
Autonomous learning skills	Setting goals 3	10	(Phuong and Tham, 2022)
	Learning strategies 4		
	Self-management skill 3		

#### 3.3.2 English speaking proficiency test

English SP was evaluated using a simulated task adapted from the College English Test-Spoken English Test (CET-SET), a nationally recognized and validated assessment for Chinese university students (Zhang and Elder, 2009). The assessment consisted of three components: A personal introduction on daily topics such as family and interests, an individual presentation lasting 1.5 min followed by a 3-min group discussion, and follow-up questions requiring the expression of opinions. All spoken responses were audio-recorded and independently rated by two experienced English language instructors, each with more than 5 years of CET-SET assessment experience.

Raters used a standardized analytic rubric targeting three latent dimensions of speaking proficiency: Accuracy (grammar and vocabulary), Fluency (speech rate and coherence), and Flexibility (adaptability and pragmatic appropriateness). Each dimension was scored on a scale from 0 to 10. Inter-rater reliability was high (ICC = 0.92 for Accuracy, 0.89 for Fluency, 0.90 for Flexibility). Any discrepancies of more than 1 point were resolved through discussion. In the structural equation SEM analysis, the three rated dimensions SP, Accuracy (SPA), SP Fluency (SPFu), and SP Flexibility (SPFe) served as observed indicators of the latent variable SP, reflecting the multidimensional nature of spoken language ability (Luoma, 2004). This methodology enabled the analysis to represent SP as a unified yet multifaceted construct.

### 3.4 Data analysis

SEM was selected as it allows for the simultaneous testing of a theoretically specified model that includes direct and indirect (mediating) relationships among latent constructs (Byrne, 2016). The model specification, including the second-order structure for EFL Learners’ Identity and the hypothesized mediation paths, was grounded in Identity Theory and prior empirical research, making SEM a confirmatory and appropriate method for testing the proposed hypotheses. Confirmatory factor analysis was employed to assess the individual constructs within the instrument by evaluating the model fit for each construct (Greene et al., 2023).

### 3.5 Confirmatory factor analysis

A total of 395 questionnaires were collected, of which five were excluded due to incomplete responses. After screening for missing data, outliers, and normality, 392 valid responses were retained for further analysis. Confirmatory factor analysis was conducted to assess the reliability and construct validity of the measurement model. Following established methodological guidelines (Fornell and Larcker, 1981; Hu and Bentler, 1999), this study examined standardized factor loadings, composite reliability, average variance extracted, and overall model fit indices.

#### 3.5.1 Convergent validity (CFA, CR, AVE)

The CFA results indicated an acceptable fit for the second order measurement model. As shown in Table 3, IDB and IDE loaded significantly on the higher order identity construct (ID), with standardized loadings of 0.742 and 0.788, respectively (*p* < 0.001). All indicators loaded significantly on their respective factors, with standardized loadings ranging from 0.708 to 0.884. Composite reliability (CR) values ranged from 0.739 to 0.865 and average variance extracted (AVE) values ranged from 0.586 to 0.681, meeting recommended thresholds (CR ≥ 0.70; AVE ≥ 0.50) and supporting convergent validity.

**TABLE 3:** Overall confirmatory factor analysis results.

Path			S.E.	C.R.	P	Standardized factor load	Squared multiple correlation (SMC)	1-SMC	Composite reliability (CR)	Average variance extracted (AVE)
IDB	< -	ID	0.132	8.727	***	0.742	0.551	0.449	0.739	0.586
IDE	< -	ID				0.788	0.621	0.379		
SAC	< -	SA	0.109	8.604	***	0.810	0.656	0.344	0.748	0.597
SAF	< -	SA				0.734	0.539	0.461		
SPA	< -	SP	0.055 0.060	14.371 14.747	*** ***	0.884	0.781	0.219	0.825	0.614
SPFe	< -	SP				0.718	0.516	0.484		
SPFu	< -	SP				0.738	0.545	0.455		
GS	< -	ALS				0.826	0.682	0.318	0.865	0.681
LS	< -	ALS	0.087	11.686	***	0.809	0.655	0.345		
ALSM	< -	ALS	0.082	11.473	***	0.840	0.706	0.294		
IDB1	< -	IDB	0.067	15.095	***	0.741	0.549	0.451	0.870	0.573
IDB2	< -	IDB				0.793	0.630	0.370		
IDB3	< -	IDB	0.070	15.184	***	0.798	0.637	0.363		
IDB5	< -	IDB	0.070	13.773	***	0.724	0.525	0.475		
IDB6	< -	IDB	0.078	13.762	***	0.724	0.524	0.476		
IDE1	< -	IDE				0.817	0.667	0.333	0.915	0.682
IDE2	< -	IDE	0.050	19.742	***	0.852	0.725	0.275		
IDE3	< -	IDE	0.047	19.617	***	0.848	0.719	0.281		
IDE4	< -	IDE	0.049	18.505	***	0.814	0.662	0.338		
IDE5	< -	IDE	0.050	18.010	***	0.798	0.637	0.363		
SAC1	< -	SAC	0.065	14.152	***	0.768	0.591	0.409	0.852	0.590
SAC2	< -	SAC				0.732	0.535	0.465		
SAC3	< -	SAC	0.065	15.584	***	0.805	0.648	0.352		
SAC4	< -	SAC	0.067	14.831	***	0.765	0.586	0.414		
SAF1	< -	SAF	0.058	15.706	***	0.784	0.615	0.385	0.913	0.637
SAF2	< -	SAF				0.746	0.556	0.444		
SAF3	< -	SAF	0.059	17.649	***	0.819	0.671	0.329		
SAF4	< -	SAF	0.056	18.406	***	0.847	0.717	0.283		
SAF5	< -	SAF	0.057	16.249	***	0.767	0.588	0.412		
SAF6	< -	SAF	0.059	17.758	***	0.823	0.678	0.322		
GS1	< -	GS	0.059	16.998	***	0.833	0.694	0.306	0.836	0.631
GS2	< -	GS				0.816	0.667	0.333		
GS3	< -	GS	0.054	15.072	***	0.730	0.533	0.467		
LS1	< -	LS	0.051	17.739	***	0.839	0.703	0.297	0.862	0.611
LS2	< -	LS				0.804	0.646	0.354		
LS3	< -	LS	0.051	16.786	***	0.770	0.593	0.407		
LS4	< -	LS	0.052	15.065	***	0.708	0.501	0.499		
ALSM1	< -	ALSM				0.799	0.638	0.362	0.828	0.617
ALSM2	< -	ALSM	0.088	16.054	***	0.814	0.662	0.338		
ALSM3	< -	ALSM	0.070	14.682	***	0.742	0.551	0.449		

The dimensions of Speaking Proficiency (SP) include Speaking Proficiency Accuracy (SPA), Speaking Proficiency Flexibility (SPFe), and Speaking Proficiency Fluency (SPFu). The components of Autonomous Learning Skills (ALSs) include Goal Setting (GS), Learning Strategies (LS), and Self-Management Skills (ALSM). These abbreviations represent key constructs in the study, with each dimension reflecting a specific aspect of the learners’ proficiency and autonomous learning abilities. ***Indicates a *p*-value <0.001, representing statistical significance at the 0.1% level.

The Squared Multiple Correlation (SMC) values, which indicate the extent to which the latent variables explain variance, ranged from 0.501 to 0.781. This means the latent variables accounted for a large portion of the variance in the observed variables. The residual variance (1-SMC) ranged from 0.219 to 0.499, suggesting the model explained most of the variance. Overall, these results confirm that the measurement constructs in this study are reliable and valid, supporting the use of this measurement model in further analyses.

#### 3.5.2 Discriminant validity

Discriminant validity was assessed to ensure that the constructs in the model are distinct. Following the Fornell and Larcker (1981) criterion, the square root of the average variance extracted (√AVE) for each construct was compared with the absolute value of its correlations with other constructs. As shown in Table 4, the √AVE values (diagonal) are higher than the absolute inter construct correlations (off diagonal), supporting discriminant validity.

**TABLE 4:** Discriminant validity assessment (fornell-larcker criterion).

Construct	1	2	3	4
1. Identity (ID)	0.766	0.773	0.784	0.825
2. Speaking anxiety (SA)	−0.492			
3. Speaking proficiency (SP)	0.534	−0.418		
4. Autonomous learning (ALS)	0.631	−0.502	0.457	

### 3.6 Structural model and mediation analysis

After establishing an acceptable measurement model, the structural model was estimated to test the hypothesized relationships among identity, SA, SP, and ALS. Direct effects were evaluated using standardized path coefficients. The mediating role of SA was examined by estimating the indirect effects of ID on SP and ALS via SA. Indirect effects were tested using bootstrapped 95% confidence intervals, and mediation was considered significant when the confidence interval did not include zero. Structural model fit was evaluated using χ^2^/df, CFI, TLI, RMSEA, SRMR, and AGFI.

### 3.7 Ethical considerations

The Ethics Committee of Universiti Sains Malaysia approved this study (Protocol code: USM/JEPeM/PP/24100950) because it involved human data. Participating students signed informed consent forms. All participants were clearly told about the research purpose and procedures, and gave written consent before taking part. Participation was voluntary, and we kept all data anonymous and confidential throughout the study.

## 4 Results and discussion

**FIGURE 2:**
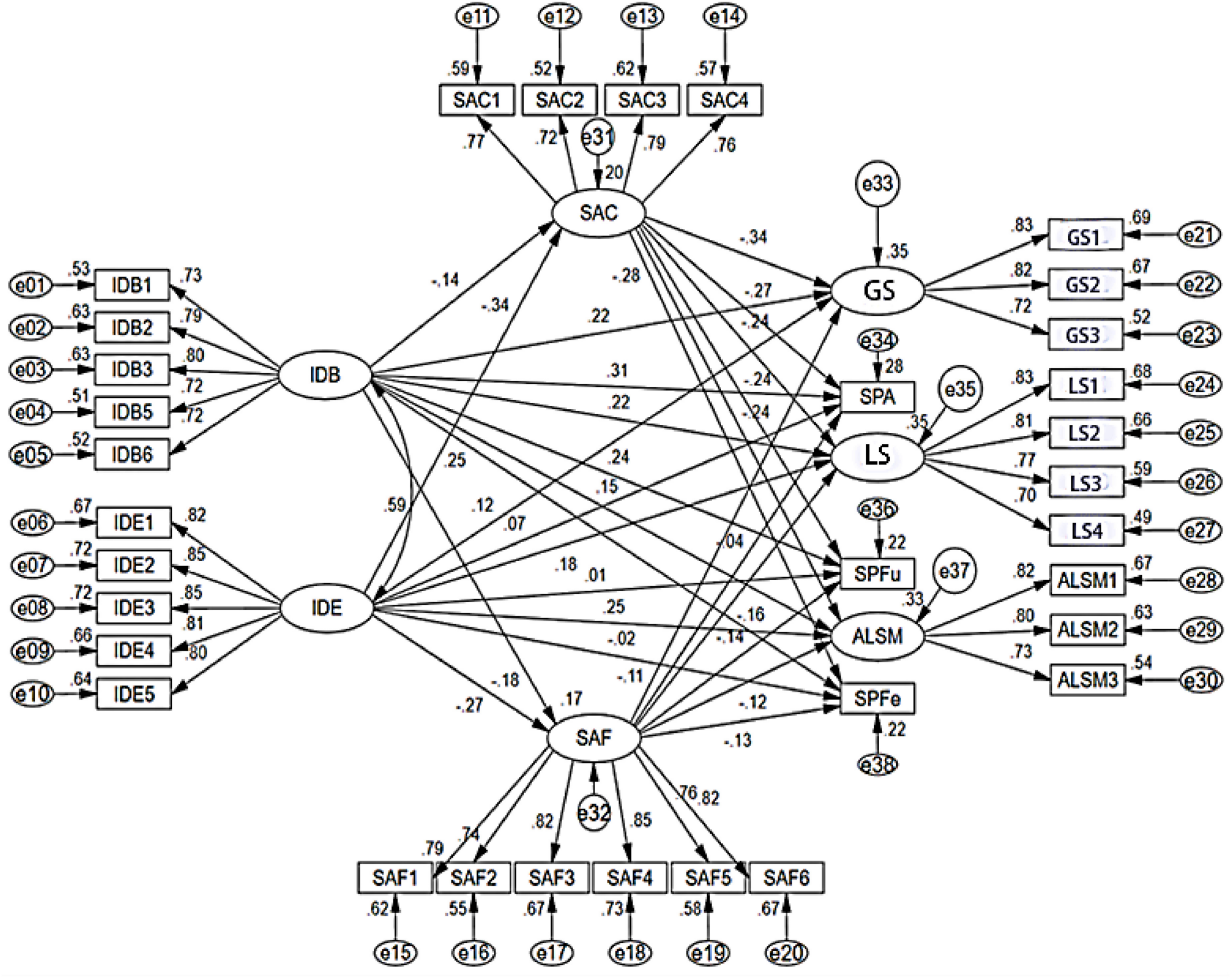
Structural equation model path analysis.

This study looked at how EFL learners’ identity affects English speaking proficiency and autonomous learning skills among non-English major university students in China, with speaking anxiety as a mediating factor. The data were analyzed using structural equation modeling, and the model fit indices showed a good fit: χ^2^/df = 2.581, RMSEA = 0.064, SRMR = 0.079, AGFI = 0.810, TLI = 0.901, and CFI = 0.900. These values meet the standard thresholds for SEM research. Figure 2 shows the results of the path analysis, including the hypothesized relationships among EFL learners’ identity, speaking anxiety, English speaking proficiency, and autonomous learning skills. The figure also presents the first-order dimensions of each latent construct.

### 4.1 H1: EFL learners’ identity has a significant influence on English speaking proficiency

The results presented in Figure 2 provide empirical support for H1, indicating that EFL learners’ identity significantly predicts SP. Consistent with the second order specification, the structural paths show that the higher order identity construct, as indicated by IDB and IDE, is positively associated with SP. In other words, students with a stronger overall learner identity tended to demonstrate better speaking performance across the assessed facets of accuracy, fluency, and flexibility. At the measurement level, both first order dimensions, IDB and IDE, loaded significantly on the higher order identity factor, as reported in section 3.4 and Table 3. These results support the conceptualization of learner identity as an integrated construct within the structural equation modeling framework.

### 4.2 H2: EFL learners’ identity has a significant influence on autonomous learning skills

As shown in Figure 2, H2 posited that EFL learners’ identity would significantly influence ALS, including GS, LS, and ALSM. The results support H2: the second-order Identity construct, indicated by IDB and IDE, demonstrated a significant positive relationship with ALS and its dimensions. This suggests that students who experience a stronger overall sense of identity as EFL learners are more likely to set learning goals, apply learning strategies, and manage their learning processes effectively.

### 4.3 H3: the mediating role of speaking anxiety

The mediating role of SA was evaluated within the structural model. Based on the theoretical framework that conceptualizes EFL Learners’ Identity as a cohesive higher-order construct, comprising the correlated dimensions of IDB and IDE, and supported by confirmatory factor analysis (see section 3.4), the mediation analysis utilized the second-order ID construct. This method aligns with the objective of examining the overall mechanism by which an integrated sense of identity influences outcomes, rather than analyzing potentially collinear dimensions separately. It also offers a more parsimonious assessment of the theoretical model (Chen et al., 2005). Therefore, the paths from the individual first-order dimensions (IDB, IDE) to the mediator and outcomes were constrained to be accounted for by their shared variance, as represented by the second-order factor.

The mediating role of SA was evaluated using the bootstrap method. A total of 5,000 bootstrap samples were drawn from the original dataset (*N* = 392), producing 1,000 estimates of the mediating effect to approximate the sampling distribution. The 95% confidence interval was calculated from the 2.5th and 97.5th percentiles of these estimates. Mediation was considered significant because the confidence interval did not include zero. The bootstrapped mediation analysis demonstrated that SA, including SAC and SAF, significantly mediated the relationship between EFL learners’ ID and both SP and ALS, as illustrated in Figure 2.

**FIGURE 3:**
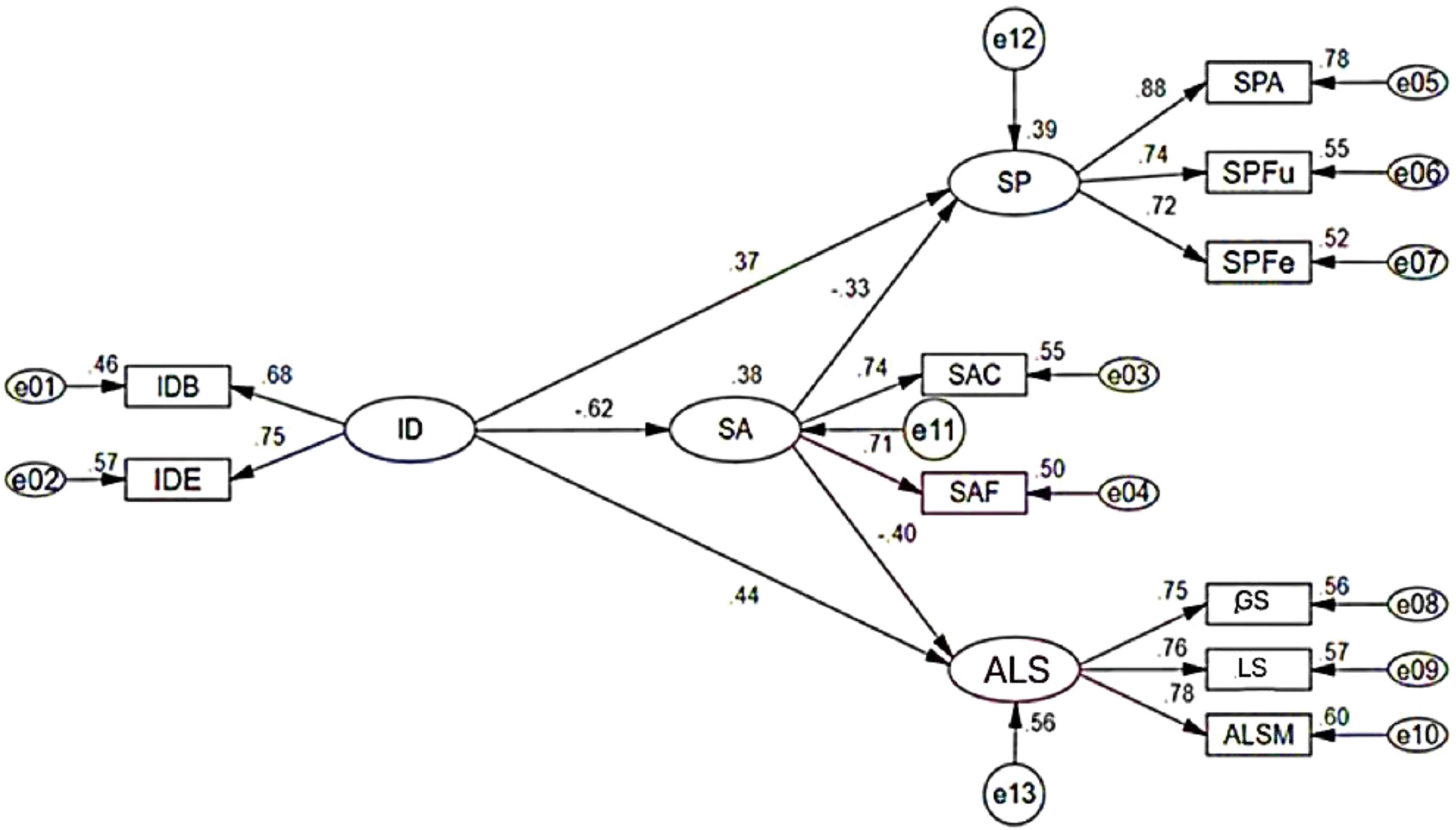
Mediation effect of SA.

Figure 3 shows the path diagram for the structural equation model with standardized estimates. It shows that SA acts as a mediator between the second-order EFL Learners’ identity and the outcome variables, SP and ALS. The first-order dimensions of identity, IDB, and IDE are included in the higher-order factor.

Table 5 reports the results of the bootstrap test examining the mediating effect of SA on the relationships between identity and SP and between identity and ALS. The indirect effect of identity on SP through SA was 0.202 (95% confidence interval: 0.055, 0.402), while the direct effect was 0.367 (95% confidence interval: 0.093, 0.588). Both effects reached statistical significance. The indirect effect constituted 35.5% of the total effect (0.569). Regarding ALS, the indirect effect of identity via SA was 0.244 (95% CI: 0.098, 0.413), and the direct effect was 0.436 (95% CI: 0.189, 0.683). The indirect effect represented 35.9% of the total effect (0.680). These results indicate that SA partially mediates the influence of EFL learners’ identity on both SP and ALS, accounting for 35.5 and 35.9% of the total effects, respectively. These findings support H3, highlighting the significant mediating role of SA in the relationship between EFL learners identify and both SP and ALS.

**TABLE 5:** Bootstrap test results of the mediating effect of SA.

Path	Beta	SE	*p*	95% CI bootstrap (BC)	
				Lower	Upper
Standardized total effects ID→SP	0.569	0.062	0.000	0.443	0.685
Standardized direct effects ID→SP	0.367	0.128	0.014	0.093	0.588
Standardized indirect effects ID→SA→SP	0.202	0.092	0.014	0.055	0.402
Standardized total effects ID→ALS	0.680	0.065	0.000	0.548	0.808
Standardized direct effects ID→ALS	0.436	0.127	0.003	0.189	0.683
Standardized indirect effects ID→SA→ALS	0.244	0.082	0.009	0.098	0.413

## 5 Discussion

### 5.1 The influence of EFL learners’ identity on English speaking proficiency

The findings from the structural model provide a nuanced understanding of how the distinct dimensions of EFL learners’ identity influence English SP. The results confirm the hypothesis that identity significantly predicts SP, but critically reveal that this influence is almost entirely carried by the belongingness dimension. IDB demonstrated a significant positive effect on all three assessed facets of SP accuracy, fluency, and flexibility. This robust relationship suggests that a student’s sense of connection to, and membership within, an English-using community directly facilitates their oral performance. A strong sense of belonging likely lowers psychological barriers, increases willingness to communicate, and provides a safe context for practice, thereby enhancing all dimensions of spoken output.

Contrary to theoretical expectations, IDE did not demonstrate a significant direct effect on any of the SP dimensions in this model. This result is important for refining theoretical frameworks (Tao et al., 2025; Krashen, 1982). The findings indicate that although learners may possess future-oriented aspirations regarding their English use, such expectations alone do not directly lead to improved speaking skills within the immediate learning context (Mohammadi and Izadpanah, 2019; Swain, 2005).

The non-significant result for IDE warrants further examination. Expectations may primarily function as distal motivational drivers, with their effects fully mediated by more immediate factors such as anxiety, engagement, or practice frequency, which were not fully represented in the direct paths of this model (Iloponu and Hanafi, 2025; Stets and Serpe, 2013). Alternatively, this outcome may indicate a misalignment between students’ idealized expectations and the actual opportunities or feedback available in their academic environment, thereby limiting the activating potential of these expectations. These results build on earlier theoretical work in important ways. They provide strong evidence for Identity Theory (Stets and Serpe, 2013) by showing that the social and belonging aspects of identity play a key role in performance. At the same time, the findings support Swain’s (2005) Output Hypothesis.

A secure identity, supported by IDB, seems to create the right emotional conditions for learners to develop accuracy, fluency, and flexibility. We also found that while accuracy was relatively high, there was still room to improve fluency and flexibility. This highlights a common issue in EFL classrooms, where teaching often focuses more on form than on communication. Our analysis suggests that building up IDB could help address this gap. Therefore, the primary practical implication is clear and evidence-based: pedagogical interventions aimed at boosting speaking proficiency should prioritize cultivating a classroom community that fosters a strong sense of belonging.

### 5.2 The influence of EFL learners’ identity on autonomous learning skills

This study used SEM to examine how the identity of English learners who are not English majors relates to their ALSs. The results show that identity has a clear impact on ALSs, but the strength of this effect depends on the specific aspect of identity. A sense of belonging had a strong positive effect on all three ALS components, which supports the idea that feeling connected in a learning community helps learners manage their own learning. On the other hand, learners’ expectations about teachers and the learning environment had a weaker and less consistent effect. These expectations influenced LS and ALSM, but did not affect GS.

The results show how different aspects of identity influence learner autonomy. Identity-based IDB had a clear positive effect on ALS, with a standardized coefficient of 0.216 (*p* = 0.002), highlighting its important role in supporting learner autonomy. In contrast, identity-based IDE showed a positive correlation (β = 0.124, *p* = 0.076), but this was not statistically significant at the 0.05 level. This suggests IDE might have a moderate effect, possibly depending on how well teacher and student expectations align. These findings are consistent with earlier research, such as Rachmajanti et al. (2021) and Zolfaghari and Rashidi (2023), which found that a strong classroom community identity encourages proactive learning. However, when learners’ expectations do not match classroom realities, it can make it harder for them to become autonomous learners. Unlike previous studies, this research adds to the discussion by showing how different aspects of both identity and autonomy matter.

These results support Liao et al.’s (2024) claim that a positive learner identity encourages engagement. They also reflect Wang et al.’s (2024) study on how academic self-efficacy, professional commitment, and learning autonomy are connected, and highlight the importance of differences in learners’ abilities for autonomous learning. Including SA as a mediating variable adds depth to the analysis. This approach is similar to that of Derakhshan et al. (2020), who noted that SA can limit self-directed learning.

### 5.3 Mediation of speaking anxiety

This study establishes SA as a critical mediator in the relationships between EFL learners’ identity and both SP and ALSs. The bootstrap analysis confirmed a significant indirect effect, with SA accounting for 35.5% of identity’s total effect on SP and 35.9% on ALS. This robust mediation underscores that a substantial portion of identity’s influence is channeled through the affective filter of SA, revealing a key psychological pathway. The findings position SA as a central conduit. Specifically, the results demonstrate a negative mediation where a stronger overall learner identity, primarily driven by its (IDB) dimension, is associated with reduced SA, which in turn facilitates higher SP and stronger ALS.

A positive learner identity can help reduce harmful emotional states. This study’s main theoretical contribution is to show how different parts of identity play distinct roles (Namaziandost et al., 2024). The data show that IDB directly lowers SA, but IDE does not have a significant direct effect on SA or the outcome variables (Jeong et al., 2025; Wang et al., 2021).

This divergence suggests that the emotional and affiliative component of identity (belongingness) is more directly potent in managing anxiety than future-oriented expectations, a nuance that adds specificity to identity theory in SLA. By empirically linking identity to ALS through SA mediation, this study expands the understanding of anxiety’s impact. It reveals that SA does not only impair real-time performance but also erodes the capacity for self-directed learning. Practically, these findings argue for pedagogical interventions that foster a sense of belonging, as this facet of identity is directly linked to lower anxiety and, consequently, to better proficiency and stronger ALS.

## 6 Conclusion

This study provides empirical evidence that EFL learner identity, particularly its belongingness dimension, significantly influences speaking proficiency and autonomous learning skills, both directly and through the mediating role of speaking anxiety. By identifying SA as a critical psychological pathway, the findings offer a more nuanced, mechanism-based understanding of how affective and identity-related factors jointly shape learning outcomes in the Chinese university EFL context. While the findings illuminate important relationships, their generalizability is constrained by the specific sample of sophomore non-English majors from selected Chinese public universities. Future research involving more diverse cohorts and longitudinal designs could further validate and extend these insights. Furthermore, the significant mediating role of anxiety suggests it is a key point of intervention, though its operation is likely influenced by unmeasured contextual factors such as classroom climate or instructional practices.

Theoretically, this study contributes by integrating identity theory with affective models of language learning, demonstrating their interconnectedness. Practically, it highlights the value of pedagogical approaches that deliberately foster a supportive, identity-affirming learning community to reduce anxiety and empower autonomous learning. These strategies should be viewed as complementary components within a holistic pedagogy, rather than universal solutions, calling for educators to adapt these insights thoughtfully to their specific institutional and cultural settings.

## 7 Limitations and future research

This study has several limitations. First, the data came only from sophomore non-English majors at three public universities in northeastern Guangdong, China, so the results may not apply to all Chinese university students. Second, because the study adopted a cross-sectional design, causal inferences and temporal ordering among identity, SA, SP, and ALS cannot be established. Finally, other contextual variables (e.g., instructional practices or broader sociocultural factors) were not modeled and may also influence SP and ALS.

Future research should address these limitations and build on these findings. Long-term or experimental studies are needed to show cause and effect and to see how identity and anxiety change over time. Using different methods and drawing on more sources would make the results more reliable. To see if the model holds up, researchers should repeat the study with different groups, such as English majors or students from other regions or countries. Finally, these results suggest that intervention research could be useful. For example, experimental studies could test whether specific teaching programs help students feel they belong and reduce their speaking anxiety, and whether these changes improve proficiency and autonomy. This would help turn these findings into practical strategies.

## Data Availability

The original contributions presented in the study are included in the article/Supplementary material, further inquiries can be directed to the corresponding author.
