# Supplementary material for: The effect of EFL learners’ identity on English speaking proficiency and autonomous learning skills among university students: the mediating role of speaking anxiety
Source: Front Psychol. 2026 Apr 10;17:1773756. doi: 10.3389/fpsyg.2026.1773756 (PMC13106447; doi:10.3389/fpsyg.2026.1773756)
Supplement: Supplementary file 1 [file Supplementary_file_1.pdf]

Appendix A

Figure A1. Evaluation Scale.

Dear Participants,

First of all, thank you for taking the time to fill out this questionnaire so that this research can proceed smoothly. I am extremely grateful! This is an academic questionnaire. Your valuable opinions will help us conduct precise research on this topic. This questionnaire will be anonymous. All personal information will only be used for analysis and will never be released to the public. Please feel free to fill it out. There are no standard answers to the questions in the questionnaire. You only need to fill in the answers according to your actual situation and feelings. Finally, thank you again for your support and cooperation, and I am deeply grateful for your sincere efforts!

Part 1: Basic Demographic Information

1. Gender: □Male □Female
2. You come from: □rural areas □towns
3. Subject Categories: □Humanities and Social Sciences □Engineering □Science □Medicine □Agronomy □Others
4. Grade: □Freshman □Sophomore □Junior students □Senior students

Part 2: EFL Learners' Identity Scale

The following is a description of **EFL Learners' Identity**. You can only choose one answer for each question. Your answer will be kept confidential.

"1"=Strongly disagree, "2"=Basically disagree, "3"=Not sure,

"4"=basically agree, "5"=Strongly agree.

		**EFL Learners' Identity Scale**	1	2	3	4	5
Belongingness	1	I feel a sense of belonging to the language institute where I am learning English.					
	2	I see myself as a member of the English language learners' community in China.					
	3	I see myself as a member of the English language community across the world.					
	5	I like to develop good relationships with my classmates in and out of the English class.					
	6	I like to develop good relationships with native English speakers.					
Expectations	1	I expect teacher in school to consider my emotional and psychological needs as an English learner.					
	2	I expect my relationships with my classmates and teachers to meet my educational needs.					
	3	I expect the textbooks and materials taught in the course to be well-matched to my needs.					
	4	I like my teacher to take my suggestions about teaching into account.					
	5	I expect my out-of-class communication with others to bring me a high level of satisfaction.					

Figure A1. (continued)

Part 3: Speaking Anxiety Scale

The following is a description of **Speaking Anxiety of English speaking**. You can only choose one answer for each question. Your answer will be kept confidential.

"1"=Strongly disagree, "2"= Basically disagree, "3"=Not sure, "4"= Basically agree, "5"=Strongly agree.

		**Speaking Anxiety Scale**	1	2	3	4	5
**Communication apprehension**	1	I start to be panic when I have to speak English without a preparation in advance.					
	2	In a speaking class, I can get so nervous I forget things I know.					
	3	I get nervous and confused when I am speaking English					
	4	I dislike using my voice and body expressively while I am speaking English.					
**Fear of Negative Evaluation**	1	I am afraid that other students will laugh at me while I am speaking English.					
	2	I can feel my heart pounding when I am going to be called on.					
	3	It embarrasses me to volunteer to go out first to speak English.					
	4	Certain parts of my body feel very tense and rigid while I am speaking.					
	5	I feel anxious while I am waiting to speak English.					
	6	I have trouble to coordinate my movements while I am speaking English.					

Part 4: Autonomous learning skills Scale (students' self-evaluation)

The following is a description of **Autonomous learning skills**. You can only choose one answer for each question. Your answer will be kept confidential.
"1"=Strongly disagree, "2"=Basically disagree, "3"=Not sure, "4"=basically agree, "5"=Strongly agree.

		Autonomous learning skills Scale	1	2	3	4	5
**Setting goals**	1	I can determine clear learning objectives to achieve them.					
	2	I can set my learning plan whenever each homework is given					
	3	I can seek clarification of the learned contents on the Internet when needed					
**Learning strategies**	1	I can arrange discussions with my classmates outside class to complete assigned projects of English-speaking learning.					
	2	I can ask for help from my classmates or teacher when facing difficulties in given tasks					
	3	I can strive to complete given tasks on time and in expectation as well					
	4	I can use in class and outside class time constructively to complete homework promptly and excellently					

Figure A1. (continued)

**Self-management skill**	1	I can assess my learning capacity based on my strengths as well as weaknesses during teamwork					
	2	I can monitor my study through diary writing after each lesson					
	3	I can consistently engage in English class as I am given more freedom of ideas expression					
**Thank you!**							
